# Awareness of HPV and HPV vaccine among college students in China

**DOI:** 10.3389/fpubh.2024.1451320

**Published:** 2024-09-18

**Authors:** Manman Li, Fengzhi Zhang, Yun Shi, Kaige Shi, Xiaoxue Li, Hua Bai

**Affiliations:** ^1^Department of Gynecology, The Third Affiliated Hospital of Zhengzhou University, Zhengzhou, China; ^2^Department of Nursing, The Third Affiliated Hospital of Zhengzhou University, Zhengzhou, China; ^3^Department of Infection Control, The Third Affiliated Hospital of Zhengzhou University, Zhengzhou, China

**Keywords:** human papillomavirus, vaccine, cervical cancer, willingness to vaccination, college students

## Abstract

**Background:**

Cervical cancer is the fourth most common cancer among women, HPV vaccine can reduce the incidence of cervical cancer by approximately 70%. Sexual behavior is a direct risk factor for HPV infection, and sexually active college students, therefore, receive attention for HPV vaccination. This study aimed to investigate the awareness of HPV and its vaccine among college students in Zhengzhou, and to explore the factors influencing their awareness of HPV vaccine, to understand college students’ willingness to receive the vaccine. The findings of this study will lay a foundation for cervical cancer prevention.

**Methods:**

Using a multistage random sampling method, 650 college students from four universities in Zhengzhou were selected. A self-administered questionnaire on the awareness of HPV and its vaccine, and willingness to receive HPV vaccination was carried out. Logistic regression was used to analyze the factors influencing students’ awareness of the HPV vaccine.

**Results:**

58.0% of college students had heard of HPV, and 72.8% of college students had heard of HPV vaccine. Logistic regression showed that gender, major, grade, mean monthly consumption level, sexual history, and mother cervical cancer screening participation significantly influenced the awareness of HPV vaccine (*p* < 0.05). Only 27(4.2%) college students had received the HPV vaccine. 63.2% of college students expressed their willingness to get vaccinated.

**Conclusion:**

The awareness of HPV and its vaccine among college students in Zhengzhou needs improvement. Although the vaccination rate is low, most college students are willing to be vaccinated. Diverse health education programs should be conducted for different groups to improve awareness of cervical cancer prevention and promote vaccination.

## 1 Introduction

Gynecological cancers are a common type of malignant reproductive system tumors in women. The latest statistical report from the International Agency for Research on Cancer showed that in 2022, there were an estimated 1.42 million new cases of gynecological cancers worldwide. This accounts for approximately 14.5% of all new cancer cases in women globally, with about 660,000 deaths representing 15.3% of all female cancer deaths (1). Cervical cancer is the fourth most common cancer among women, and the age of onset is trending toward a younger age. A total of 661,021 new cases and 348,189 deaths from cervical cancer annually have been recorded, with approximately 6.8% of new cases and 8.1% of deaths occurring in the world (1). China had more than 258,000 new cases of gynecological cancers in 2022, of which 111,820 were cervical cancers, accounting for 43.2% of gynecological cancers, making it the country with the highest incidence of cervical cancer worldwide (2). Cervical cancer poses a considerable threat to women’s health and imposes a substantial economic burden on society and families, especially because the age of onset is trending toward a younger age. Therefore, accelerating the elimination of cervical cancer has become a global public health strategy (3). Up to 99% of cervical cancer cases are associated with high-risk human papillomavirus (HPV). Although most HPV infections resolve spontaneously without causing any symptoms, persistent infections can lead to cervical cancer in women (4, 5). HPV vaccines can reduce the incidence of cervical cancer by approximately 70% and prevent other diseases, such as anal cancer, genital warts, and oropharyngeal cancer (6, 7). As of March 2022, 60% of World Health Organization member countries had incorporated the HPV vaccine into their national immunization programs, with most being high-income countries. However, global HPV vaccine coverage remained low, with only 12% of women receiving a full two-dose series (8). In China, free HPV vaccination policies for eligible girls have been introduced in some cities like Jinan, Xiamen, and Wuxi, setting a positive example, however, the HPV vaccination rate still has a significant gap compared to developed countries (6). Sexual activity is a direct risk factor for HPV infection, therefore, college students have become a focus of attention (9).

This study aimed to investigate the awareness of HPV and HPV vaccine among college students in Zhengzhou, explore the factors influencing their awareness of the HPV vaccine, and understand college students’ willingness to receive the vaccine. The findings of this study will lay the foundation for cervical cancer prevention and vaccination promotion.

## 2 Materials and methods

### 2.1 Design, setting, and participants

This cross-sectional study was conducted from October to November 2020 in Zhengzhou, Henan Province, China. A stratified cluster random sampling method was used based on students’ majors and classes. At each university, one class was selected randomly according to the grade level. The study protocol was reviewed and approved by the Third Affiliated Hospital of Zhengzhou University Committee for the Protection of Human Subjects (approval no. 2021–032-01). The participants provided informed consent before enrollment in the study. All study aspects, including voluntary participation, withdrawal, study risks, confidentiality, and secured data storage, were explained.

### 2.2 Measurement

We designed a questionnaire based on expert consultations from relevant studies (9–12). The questionnaire was anonymous, and the researcher explained the purpose and significance of the study to each class and collected it promptly with the assistance of a counselor. A preliminary survey was conducted before the formal investigation, and the final questionnaire was completed after modification. The questionnaire included participants’ demographic characteristics, awareness of HPV and HPV vaccine, and whether they had received the HPV vaccine. The demographic characteristics included age, major, grade, place of residence, education level, average monthly consumption level, etc.

### 2.3 Data collection

Three research assistants were hired to collect data. Before the formal investigation, they were trained on the study protocol, and the principal researcher explained the questionnaire items. All research assistants passed the exam and were allowed to participate. Trained investigators conducted the study and collected the questionnaires on-site. Any missing or incorrect entries were promptly supplemented or corrected to ensure the validity and completeness of the responses. A total of 650 questionnaires were distributed and collected, with an effective response rate of 100%.

### 2.4 Statistical analysis

Data were analyzed using SPSS v22.0 software (IBM Corp., Armonk, NY, United States). Measurement data were expressed as mean ± standard deviation ( X ± S), and count data were expressed as frequency and percentage (%). Factors influencing college students’ awareness of HPV and the vaccine were analyzed using the chi-square test. Logistic regression was used for multiple covariate analysis to investigate the factors influencing college students’ awareness of the HPV vaccination. A *p*-value of <0.05 indicated statistical significance.

## 3 Results

### 3.1 Demographic characteristics

A total of 650 questionnaires were distributed, and all were completed, with a response rate of 100%. The mean age of the participants was 19.71 ± 1.25, and 548 (84.3%) college students were females. In addition, 332 (51.1%) were medical students and 318 (48.9%) were nonmedical students. The demographic characteristics of the participants are summarized in Table 1.

**Table 1:** Demographic characteristics of the participants (*n* = 650).

Variable	Categories	Frequency(n)	Percent (%)
Gender	Male	102	15.7
	Female	548	84.3
Major	Medical	332	51.1
	Non-medical	318	48.9
Grade	Freshman	198	30.5
	Sophomore	170	26.2
	Junior	170	26.2
	Senior	112	17.2
Place of household registration	Rural	421	64.8
	Urban	229	35.2
Education level	College and below	278	42.8
	Undergraduate and above	372	57.2
Mean monthly consumption level (yuan/month)	<1,000	172	26.5
	1,000 ~ 1,999	409	62.9
	>2,000	69	10.6
Cancer history of family members or friends	Yes	121	18.6
	No	529	81.4
Sexual history	Yes	102	15.7
	No	548	84.3
Mother’s participation in cervical cancer screening	Never	152	23.4
	Occasional	89	13.7
	Regular	47	7.2
	Unclear	362	55.7

### 3.2 Awareness of HPV among college students

A total of 377 (58%) college students had heard of HPV. Of these participants, 52% were males and 59.1% were females, with no statistically significant difference (*p* > 0.05). Statistically significant differences existed in the awareness of HPV among the different groups regarding major, grade, place of household registration, education level, mean monthly consumption level, sexual history, and mother’s participation in cervical cancer screening (*p* < 0.05). Detailed information is provided in Table 2.

**Table 2:** Univariate analysis for the awareness of HPV among college students [(n)%].

Variable	Categories	Have you heard of HPV?		χ^2^	*p*-value
		Yes	No		
Gender	Male	53(52.0)	49(48.0)	1.811	0.178
	Female	324(59.1)	224(40.9)		
Major	Medical	228(68.7)	104(31.3)	31.744	<0.001
	Non-medical	149(46.9)	149(46.9)		
Grade	Freshman	71(35.9)	127(64.1)	66.355	<0.001
	Sophomore	105(61.8)	65(38.2)		
	Junior	112(65.9)	58(34.1)		
	Senior	89(79.5)	23(20.5)		
Place of household registration	Rural	226(53.7)	195(46.3)	9.148	0.002
	Urban	151(65.9)	151(65.9)		
Education level	College and below	139(50.0)	139(50.0)	12.762	<0.001
	Undergraduate and above	238(64.0)	134(36.0)		
Mean monthly consumption level (yuan/month)	<1,000	80(46.5)	92(53.5)	19.804	<0.001
	1,000 ~ 1,999	244(59.7)	165(40.3)		
	>2,000	53(76.8)	16(23.2)		
Cancer history of family members or friends	Yes	75(62.0)	46(38.0)	0.968	0.325
	No	302(57.1)	227(42.9)		
Sexual history	Yes	73(71.6)	29(28.4)	9.144	0.002
	No	304(55.5)	244(44.5)		
Mother’s participation in cervical cancer screening	Never	102(67.1)	50(32.9)	51.759	<0.001
	Occasional	70(78.7)	19(21.3)		
	Regular	38(80.9)	9(19.1)		
	Unclear	167(46.1)	195(53.9)		

### 3.3 Univariate analysis for the awareness of HPV vaccine among college students

The associations between the participants’ characteristics and awareness of HPV vaccine are shown in Table 3. A total of 473 (72.8%) college students had heard about the HPV vaccine. Significant differences were observed in the awareness of HPV vaccine between different groups in terms of gender, major, grade, place of household registration, mean monthly consumption level, sexual history, and mother’s participation in cervical cancer screening (*p* < 0.05). No statistically significant differences were found in education level and cancer history of family members or friends (*p* > 0.05).

**Table 3:** Univariate analysis for the awareness of HPV vaccine among college students[(n)%].

Variable	Categories	Have you heard of HPV vaccine?		χ^2^	*P*-value
		Yes	No		
Gender	Male	58(56.9)	44(43.1)	15.448	<0.001
	Female	415(75.7)	133(24.3)		
Major	Medical	260(78.3)	72(21.7)	10.526	0.001
	Non-medical	213(67.0)	105(33.0)		
Grade	Freshman	100(50.5)	98(49.5)	74.767	<0.001
	Sophomore	133(78.2)	37(21.8)		
	Junior	141(82.9)	29(17.1)		
	Senior	99(88.4)	13(11.6)		
Place of household registration	Rural	293(69.6)	128(30.4)	6.072	0.014
	Urban	180(78.6)	49(21.4)		
Education level	College and below	194(69.8)	84(30.2)	2.184	0.139
	Undergraduate and above	279(75.0)	93(25.0)		
Mean monthly consumption level (yuan/month)	<1,000	106(61.6)	66(38.4)	21.610	<0.001
	1,000 ~ 1,999	305(74.6)	104(25.4)		
	>2,000	62(89.9)	7(10.1)		
Cancer history of family members or friends	Yes	96(79.3)	25(20.7)	3.238	0.072
	No	377(71.3)	152(28.7)		
Sexual history	Yes	89(87.3)	13(12.7)	12.812	<0.001
	No	384(70.1)	164(29.9)		
Mother’s participation in cervical cancer screening	Never	129(84.9)	23(15.1)	54.843	<0.001
	Occasional	80(89.9)	9(10.1)		
	Regular	42(89.4)	5(10.6)		
	Unclear	222(61.3)	140(38.7)		

### 3.4 Logistic regression with multiple covariates for the awareness of HPV vaccine among college students

To identify factors influencing awareness of HPV vaccine after adjusting for confounders among college students in Zhengzhou, logistic regression analysis was performed with a dichotomous variable (Table 4). The binary dependent variable was whether the college student had heard of the HPV vaccine, with 0 indicating “No” and 1 indicating “Yes.” Independent variables included gender, major, grade, place of household registration, mean monthly consumption level, sexual history, and mother’s participation in cervical cancer screening (inclusion criteria *α* = 0.05, exclusion criteria *β* = 0.10). The results showed that gender, major, grade, mean monthly consumption level, sexual history, and mother’s participation in cervical cancer screening entered the regression model. The overall model was significant (*χ*^2^ = 170.513, *p* < 0.001). The Hosmer–Lemeshow test indicated good model fit (*χ*^2^ = 11.765, *p* = 0.162).

**Table 4:** Logistic regression with multiple covariates for the awareness of HPV vaccine among college students.

Variable	Categories	B	*P*-value	OR	95%CI
Gender	Female	1.221	<0.001	3.390	(1.978–5.811)
Major	Non-medical	−0.957	<0.001	0.384	(0.250–0.591)
Grade	Sophomore	1.401	<0.001	4.058	(2.423–6.794)
	Junior	1.505	<0.001	4.506	(2.621–7.746)
	Senior	2.042	<0.001	7.708	(3.783–15.702)
Mean monthly consumption level (yuan/month)	1,000 ~ 1999	0.750	0.002	2.024	(1.297–3.157)
	>2000	1.755	<0.001	5.785	(2.251–14.866)
Sexual history	No	−0.966	0.007	0.381	(0.188–0.772)
Mother’s participation in cervical cancer screening	Occasional	1.009	0.029	2.742	(1.109–6.779)
	Regular	0.383	0.500	1.466	(0.482–4.461)
	Unclear	−0.737	0.008	0.479	(0.277–0.828)

### 3.5 HPV vaccination status among college students

Only 27 (4.2%) college students had received the HPV vaccination. Of the college students, 63.2% expressed willingness to receive the HPV vaccine, while 36.8% were unwilling. Among those who were unwilling, 25.7, 22.9, 22.9, and 20% cited reasons such as few people around them being vaccinated, high costs, and concerns about vaccine safety, respectively. Of the students, 48.8% believed that the cost should be shared between individuals and the government, whereas 34.0% thought that it should be covered by health insurance. A total of 404 (62.2%) college students indicated willingness to learn about the HPV vaccine through WeChat or Weibo, and 398 (61.2%) wanted to learn through promotional brochures (Table 5).

**Table 5:** Ways to learn about the HPV vaccine among college students.

Ways to learn about the HPV vaccine	Frequency (n)	Percent (%)
On-site classroom lectures	370	56.9
Online courses	348	53.5
Brochures	398	61.2
WeChat, Weibo	404	62.2
Short videos	363	55.8
Consultation with medical personnel	360	55.4
Other methods	46	7.1

## 4 Discussion

### 4.1 Awareness of HPV among college students in Zhengzhou

In this study, 58% of college students in Zhengzhou had heard of HPV, with 52.0% of males and 59.1% of females aware of it. This result is slightly higher than that reported by Shi et al. for students in North China, where 52.8% had heard of HPV, but lower than the awareness levels among international students (12–14). Nkwonta et al. conducted a health belief model-based study on college students in the United States and found that more than two-thirds of participants had heard of HPV infection (13). This difference could be attributed to regional variations, educational levels, and cultural differences. Foreign countries have conducted earlier research on cervical cancer, resulting in a broader dissemination of related prevention and treatment knowledge. Australia was among the first nations to implement a national HPV vaccination program in 2007, providing three doses of a quadrivalent vaccine to girls aged 12–13. The program was expanded in 2013 to include boys and girls (15). The United Kingdom’s national HPV immunization program launched in 2008 successfully prevented HPV infections, with most boys and girls vaccinated in schools (16). Consequently, their awareness of HPV infection is relatively high.

This study indicates that medical students have a higher level of awareness of HPV than nonmedical students. College students with higher grades, education levels, and mean monthly consumption levels also had a better understanding of HPV. Students whose mothers regularly participated in cervical cancer screening, hold urban household registrations, and have a sexual history generally possess a greater awareness of HPV. This aligns with domestic and international studies (9, 12, 13). This could be because, in general, more educated students have greater knowledge. Owing to their specialized fields, medical students can understand HPV comprehensively through textbooks, classroom lectures, internships, and other methods, leading them to be more concerned about their physical and mental health. In contrast, nonmedical students have relatively limited access to information on cervical cancer. Therefore, it is essential to promote HPV knowledge among college students and raise their awareness of disease prevention.

### 4.2 Awareness of HPV vaccines among college students in Zhengzhou

Currently, available HPV vaccines include Gardasil quadrivalent, which prevents infection with HPV types 6, 11, 16, and 18; Gardasil 9-valent, which prevents infection with HPV types 6, 11, 16, 18, 31, 33, 45, 52, and 58, and Gervarix bivalent, produced by GlaxoSmithKline, which primarily prevents infection with HPV types 16 and 18 (17, 18). In 2019, the first domestically produced, bivalent HPV vaccine was approved (19). With the widespread availability of HPV vaccines in China, various healthcare institutions, including hospitals and community health service centers, can provide vaccination services to women. This greatly enhances the accessibility for preventing cervical cancer, which has clear pathological processes and considerable real-world implications. In this study, 72.8% of college students had heard of the HPV vaccine, a higher rate than in the study by Shi et al. (12). This difference may be because of the growing awareness regarding cervical cancer prevention in recent years. Many college students are gaining knowledge about HPV vaccines through mobile phones, TV, lectures, and science education videos, which have improved their understanding.

This study highlights several key findings related to HPV vaccination awareness. Women, students with more education, and higher mean monthly consumption levels are more aware of the HPV vaccine. Medical students have a higher level of awareness about HPV vaccines than nonmedical students. Students with a history of sexual activity are more knowledgeable about the HPV vaccine than those without such a history. These findings are consistent with most studies (10, 12). College students with a sexual history are sensitively aware of the HPV vaccine in their daily lives and studies. For those whose mothers regularly participate in cervical cancer screenings, this acts as a positive factor in HPV vaccine awareness, which is likely linked to the family environment. Mothers play a crucial role in families, and their education, awareness, health beliefs, and practices significantly impact their children’s health behaviors and awareness. Acceptance of the HPV vaccine is influenced more by a mother’s perception of risks and benefits than by the child’s willingness (20). Future research should focus on males, nonmedical majors, and those with lower education levels and implement various health education models to improve their understanding of the HPV vaccine.

### 4.3 HPV vaccination status among college students

Receiving the HPV vaccine can prevent infection for at least 10 years and potentially provide even longer protection. In this study, only 4.2% of college students in Zhengzhou were vaccinated against HPV, a rate significantly lower than that in developed countries such as the United States and the United Kingdom (21–23). Denmark included the HPV vaccine in its national immunization program for children in 2009, which has proven to be highly effective in preventing cervical cancer. A nationwide study published in 2021 reported that women vaccinated before the age of 17 years had an incidence rate of 14%. In contrast, those vaccinated between 17 and 20 years had an incidence rate of 32% compared with unvaccinated women (24). The United Kingdom also introduced a government-funded school vaccination program. Before the 2019 coronavirus pandemic, HPV vaccination coverage was 80%. The pandemic disrupted school-based vaccination owing to decreased school attendance. Despite recent improvements, coverage rates have not yet returned to pre-pandemic levels (21). In the United States, HPV vaccines are primarily provided in primary care and healthcare facilities. Although the vaccination rate has reached 75% (22), considerable disparities in coverage exist because of racial, ethnic, and socioeconomic differences (23). Many low- and middle-income countries face financial and infrastructural constraints preventing the inclusion of HPV vaccines in their national immunization programs.

The Outline for Women’s Development in China (2021–2030) proposes important goals such as increasing cervical cancer screening rates and promoting HPV vaccination among eligible women (25). Although the vaccination rate among college students was low in this study, 63.2% expressed a willingness to receive the HPV vaccine and were eager to learn more through channels such as WeChat, Weibo, and brochures. Some researchers have pointed out that concerns regarding vaccine side effects and an insufficient understanding of HPV vaccines or HPV-related diseases are noteworthy barriers to vaccination (26). The World Health Organization has coined the term “vaccine hesitancy,” referring to the phenomenon in which people delay or refuse vaccination despite the availability of vaccination services (27). Therefore, future studies should focus on enhancing students’ knowledge of HPV vaccines, improving public awareness of HPV and its vaccines, and conducting multichannel educational campaigns. This will help to establish college students’ health awareness and improve the vaccination rate, ultimately working toward eliminating cervical cancer.

## 5 Conclusion

Awareness of HPV and HPV vaccine among college students in Zhengzhou needs to be improved. Differences in gender, major, grade, mean monthly consumption level, sexual history, and mothers’ participation in cervical cancer screening can influence college students’ awareness of HPV vaccine. Although the vaccination rate among college students in Zhengzhou is relatively low, more than half are willing to receive the vaccine. In future studies, hospitals and community health service centers should focus on diversified health education models and provide personalized services according to the characteristics of different groups. By leveraging traditional media, new media, and offline activities, these organizations should actively promote cervical cancer prevention knowledge to advance the vaccination process.

## Data Availability

The raw data supporting the conclusions of this article will be made available by the authors, without undue reservation.
